# Tauopathy-induced retinal dysfunction in the P301S mutant human tau transgenic mouse

**DOI:** 10.1186/1750-1326-8-S1-P57

**Published:** 2013-10-04

**Authors:** Nadia Mazzaro, Erica Barini, Michel Goedert, Maria Grazia Spillantini, Paolo Medini, Laura Gasparini

**Affiliations:** 1 Neuroscience and Brain Technologies, Istituto Italiano di Tecnologia, Genova, Italy; 2 MRC Laboratory of Molecular Biology, Cambridge, UK; 3 Cambridge Centre for Brain Repair, University of Cambridge, UK

## 

Intracellular inclusions made of microtubule-associated tau protein are a defining pathological hallmark of tauopathies, which include Alzheimer disease and familial frontotemporal dementia and parkinsonism linked to chromosome 17. Altered levels of tau protein have been detected in the retina and optic nerve of patients with glaucoma, suggesting that retina degeneration and tauopathies share similar pathogenic mechanisms. We have recently demonstrated that P301S mutant human tau (tau_P301S_) mice develop tau filamentous inclusions and axonopathy in retinal ganglion neurons (RGCs), in the absence of neuronal loss or alterations in the outer retina. Moreover, we showed that tau_P301S_ transgenic retinal explants do not respond to neurotrophic stimuli *in vitro*. Here, we investigated the impact of tau pathology on RGC physiology in living animals and neurotrophin signaling pathways *in vivo.* In anesthetized 5-month old wild type (WT) and tau_P301S_ mice, we measured RGCs activity using pattern electroretinogram (pERG), which selectively detects RGC response upon pattern light stimuli exposure. In transgenic tau_P301S_ mice the amplitude of both P1 positive and N2 negative components of pERG at saturating contrast and spatial frequency was significantly smaller than WT values. Furthermore, retinal acuity was significantly reduced in tau_P301S_ mice. Using uniform flickers of light (flash ERG), we measured the activity of the outer retina and found that outer retina response was preserved in tau_P301S_ mice. Neurotrophins, and especially brain-derived neurotrophic factor (BDNF), are important modulators of neuronal survival and function in the brain and in the visual system. We therefore investigated the BDNF signaling pathway and found that BDNF signalling was altered in tau_P301S_ transgenic retinas. Our results indicate that, in the tau_P301S_ mouse, tau pathology specifically impairs the activity of RGCs, without affecting the outer retina function and is associated with BDNF signalling alterations. Given the role of BDNF in synaptic plasticity, these data suggest that mild levels of tau pathology are sufficient to trigger significant neuronal dysfunction possibly through alteration of neurotrophic signalling. *Funded by a grant of Compagnia di San Paolo awarded to LG.*

